# Urachal Carcinoma with Divergent Glandular Enteric-Type and Squamous Differentiation Associated with Bladder Exstrophy: Case Report of an Extremely Rare Entity

**DOI:** 10.3390/reports9020100

**Published:** 2026-03-26

**Authors:** Catalin-Bogdan Satala, Gabriela Patrichi, Alina-Mihaela Gurau, Daniela Mihalache

**Affiliations:** 1 Medical and Pharmaceutical Research Center, Faculty of Medicine and Pharmacy, “Dunărea de Jos” University of Galati, 800008 Galati, Romania or stlcatalin92@yahoo.com (C.-B.S.); daniela.mihalache@ugal.ro (D.M.); 2 Department of Pathology, Clinical County Emergency Hospital Braila, 810325 Braila, Romania; 3 The Doctoral School of Medicine and Pharmacy, “George Emil Palade” University of Medicine, Pharmacy, Science and Technology, 540142 Targu Mures, Romania; 4 The School for Doctoral Studies in Biomedical Sciences, “Dunărea de Jos” University of Galați, 800008 Galati, Romania; g.alinaaa96@yahoo.com

**Keywords:** urachal carcinoma, enteric-type, squamous differentiation, divergent differentiation, biphasic tumor

## Abstract

**Background and Clinical Significance**: Urachal carcinoma (UrC) is an uncommon neoplasm derived from residual embryonic structures connecting the bladder to the umbilicus. Owing to its rarity, deep anatomic location, and histologic overlap with other glandular malignancies, accurate diagnosis remains challenging. Congenital anomalies of the lower urinary tract, including bladder exstrophy, are recognized as conditions that may predispose to malignant transformation of urachal remnants, although documented cases remain scarce. **Case presentation**: We describe the case of a 52-year-old male with bladder exstrophy and intellectual disability who presented with a progressively enlarging suprapubic mass and intermittent hematuria. Radiologic evaluation demonstrated a mass arising along the urachal tract. Surgical excision revealed a tumor composed of two morphologically distinct components: an enteric-type adenocarcinoma and a squamous carcinoma. Immunohistochemical profiling indicated urachal derivation and excluded other primary sites. **Conclusions**: This case expands the morphologic spectrum of UrC and emphasizes the diagnostic value of integrating clinical risk factors with detailed histologic and immunophenotypic assessment, particularly in tumors with mixed differentiation patterns.

## 1. Introduction and Clinical Significance

Urachal carcinoma (UrC) is a rare malignant neoplasm arising from vestigial remnants of the urachus, an embryologic structure connecting the fetal bladder to the allantois [1]. In normal development, the urachus undergoes involution and persists as the median umbilical ligament [2]; however, incomplete regression may result in residual epithelial tissue capable of malignant transformation [1,2,3]. UrC accounts for less than 1% of all bladder malignancies and represents a distinct clinicopathologic entity with unique diagnostic and prognostic implications [4].

Clinically, UrC often presents with nonspecific symptoms or is detected at an advanced stage due to its extravesical location and indolent early growth pattern. Hematuria, abdominal pain, or suprapubic mass may be observed, although many patients remain asymptomatic until significant local invasion occurs. As a result, diagnosis is frequently delayed, contributing to an aggressive clinical behavior and unfavorable outcomes associated with this tumor [5,6,7].

Histologically, the majority of UrC are adenocarcinomas, most commonly of enteric or mucinous type, reflecting intestinal differentiation [8]. Less frequent patterns include signet-ring cell carcinoma and mixed morphologies [9,10]. Divergent differentiation, such as squamous, is exceptionally rare in UrC and has been reported in only a small proportion of cases [11]. The presence of such mixed histologic features may pose diagnostic challenges, particularly in distinguishing primary UrC from primary bladder adenocarcinoma or metastatic gastrointestinal malignancies [12,13].

Several risk factors have been proposed in the pathogenesis of UrC, including chronic inflammation, infection, and congenital anomalies of the urinary tract. Among these, bladder exstrophy represents a particularly strong yet rarely documented predisposing condition [14]. Bladder exstrophy is associated with chronic mucosal exposure, persistent inflammation, and metaplastic changes, all of which may contribute to malignant transformation of urachal remnants [15]. Nevertheless, reports of UrC arising in patients with bladder exstrophy remain exceedingly uncommon [16].

Herein, we describe a rare case of UrC with enteric-type differentiation and an associated squamous carcinoma component in a male patient with bladder exstrophy and no evidence of another primary adenocarcinoma. This case highlights the diagnostic complexity of UrC with divergent differentiation and underscores the importance of integrating clinical context, histomorphology, immunohistochemistry, and established diagnostic criteria to achieve an accurate diagnosis.

## 2. Case Presentation

A 52-year-old male patient with moderate intellectual disability was admitted with a progressively enlarging suprapubic mass and intermittent hematuria. The patient had a history of bladder exstrophy diagnosed at birth and surgically treated in early childhood. According to the available medical records, the procedure included bladder closure and abdominal wall reconstruction. He reported mild pressure and pain in the suprapubic area over the past 5 months. The patient had no prior diagnosis of malignancy and no known family history of urinary tract cancers. Physical examination revealed a palpable, firm mass in the anterior wall corresponding to the bladder region.

**Figure 1:**
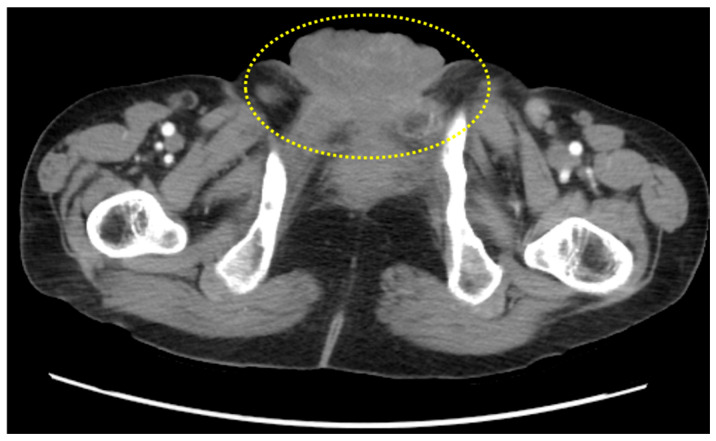
Computed tomography (CT) scan of the lower abdomen/pelvis demonstrating a midline tumor mass arising along the urachal tract.

Laboratory investigations showed mild anemia (hemoglobin 11.0 g/dL) and a slightly elevated serum creatinine (1.4 mg/dL). Tumor markers, including CEA, CA19-9, and AFP, were within normal limits. Urinalysis demonstrated microscopic hematuria. CT scan of the abdomen and pelvis revealed a heterogeneous mass, measuring approximately 6.0 × 5.5 cm, centered along the urachal remnant and extending from the bladder dome toward the umbilicus. The mass exhibited areas of soft-tissue density. No regional lymphadenopathy or distant metastases were identified (Figure 1).

The patient underwent en-bloc surgical excision, including partial cystectomy with resection of the urachal remnant. Gross examination revealed a 5.8 cm nodular mass along the urachal tract. The umbilical and peripheral bladder margins were macroscopically free of tumor. Based on the available pathologic findings, the tumor corresponded to pT3 disease according to the TNM staging system, adapted after Sheldon’s criteria. No lymph node metastases were identified. On cut surface, the tumor appeared heterogeneous, with firm white-tan regions interspersed with softer areas.

**Figure 2:**
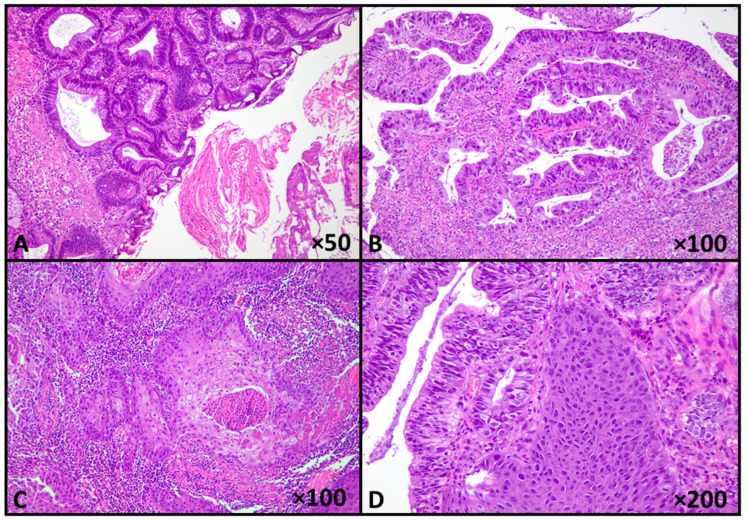
Biphasic urachal carcinoma, with enteric-type and squamous components (**A**). Enteric-type adenocarcinoma with papillary and glandular architecture, with high-grade nuclear features (**B**), and squamous carcinoma component (**C**,**D**) (Hematoxylin-Eosin stain; original magnifications shown in panels).

Microscopic examination revealed a malignant epithelial neoplasm arising in the urachal region, displaying a distinct biphasic morphology, consistent with a diagnosis of mixed urachal carcinoma: the predominant component corresponded to an enteric-type adenocarcinoma characterized by a complex glandular and papillary architectural pattern. The neoplastic glands and papillae were lined by pleomorphic epithelial cells with enlarged, hyperchromatic nuclei, irregular nuclear contours, and conspicuous nucleoli. Mitotic activity was brisk, reaching 11 mitoses per 10 high-power fields, including atypical forms. Areas of luminal necrosis and focal cribriform architecture were present. Intermixed with the glandular component, a second malignant population was identified, composed of invasive nests and cords of polygonal tumor cells with abundant eosinophilic cytoplasm, distinct cell borders, and intercellular bridges. Focal keratin pearl formation was also observed. These morphologic features support true squamous differentiation rather than squamous metaplastic change within the adenocarcinoma (Figure 2).

**Figure 3:**
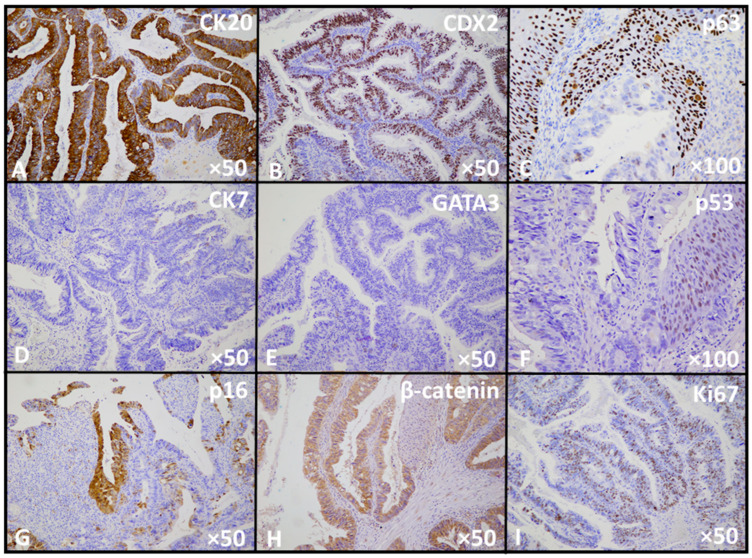
Immunophenotypic profile of the urachal carcinoma, demonstrating expression of enteric markers (**A**,**B**) and squamous markers (**C**), with absence of urothelial markers (**D**,**E**). Aberrant p53 expression with null-type pattern is observed in the glandular component (**F**), accompanied by focal block-type p16 staining (**G**). β-catenin shows predominantly membranous expression (**H**). The high proliferative activity is reflected by the increased Ki67 index (**I**).

Immunohistochemical analysis further characterized the biphasic nature of the tumor. The enteric-type adenocarcinoma component showed strong nuclear expression of CDX2 and diffuse positivity for CK20, while CK7 and GATA3 were negative. β-catenin demonstrated a predominantly membranous staining pattern without nuclear accumulation. p16 showed focal block-type (continuous cyto-nuclear) staining, whereas p53 exhibited a mutant (null-type) pattern with complete absence of nuclear expression in tumor cells. The proliferative index assessed by Ki-67 was markedly elevated, reaching approximately 70% in the glandular component. In contrast, the squamous carcinoma component, confirmed immunohistochemically by nuclear p63 positivity, lacked expression of CK20, CDX2, CK7, and GATA3. p53 staining in this area displayed a wild-type pattern. β-catenin showed focal nuclear reactivity. These findings support true divergent differentiation within a single neoplasm rather than collision of two independent tumors and, in conjunction with the tumor location and clinical findings, are consistent with primary urachal carcinoma (Figure 3).

The postoperative course was uneventful, and the patient was discharged on postoperative day 7. Due to the rarity of the tumor and the lack of standardized adjuvant therapy for UrC with squamous differentiation, the patient was referred to an oncology center abroad, with no follow-up data available.

## 3. Discussion

Urachal carcinoma (UrC) represents a distinct and uncommon malignancy arising from embryologic remnants of the urachus, characterized by unique clinical, morphologic, and biologic features that distinguish it from other primary bladder tumors [17]. The rarity of this neoplasm, combined with its often-subtle clinical presentation, has contributed to persistent challenges in diagnosis, classification, and management [18]. Most UrC are adenocarcinomas with enteric or mucinous differentiation, reflecting the glandular potential of urachal epithelium [19]; however, a broad histologic spectrum has been increasingly recognized, underscoring the biological heterogeneity of this entity [20]. Although direct invasion of the bladder mucosa was not identified histologically in the present case, hematuria may occur in patients with urachal tumors due to irritation of the adjacent bladder wall, mucosal congestion, or microscopic mucosal involvement that may not be captured in routine histologic sampling. In addition, the patient’s history of bladder exstrophy and prior reconstructive surgery may have contributed to mucosal fragility or chronic irritation, which could also represent a potential source of hematuria.

The present case is notable for the co-existence of enteric-type adenocarcinoma and a distinct squamous carcinoma component arising in the setting of bladder exstrophy, a rare congenital anomaly that confers a markedly increased risk for this type of cancer.

To contextualize the rarity of this entity, we performed a focused review of the available literature using major biomedical databases including PubMed/MEDLINE, Scopus, and Web of Science, using combinations of the keywords “urachal carcinoma”, “urachal adenocarcinoma”, “squamous differentiation”, “adenosquamous”, and “urachus”. Based on this search, only four previously reported cases of urachal carcinoma with a squamous carcinoma component were identified in the English-language literature prior to the present report. These previously published cases are summarized in Table 1 [1,11,21,22].

Bladder exstrophy creates a unique microenvironment characterized by chronic mucosal exposure, repeated irritation, and long-standing inflammation, all of which are recognized drivers of epithelial metaplastic changes and carcinogenesis [16]. In this context, urachal remnants may undergo intestinal metaplasia, providing a plausible precursor for enteric-type adenocarcinoma [23]. The additional presence of squamous differentiation suggests further phenotypic divergence, potentially reflecting metaplastic or transdifferentiation processes induced by sustained inflammatory and mechanical stress [24].

Squamous differentiation in UrC is exceptionally rare, reported in less than 5% of cases, and its biological significance remains incompletely understood. In reported cases, squamous components have ranged from focal areas of keratinizing differentiation within an otherwise typical adenocarcinoma to clearly demarcated biphasic tumors composed of glandular and squamous carcinoma elements [11,16,24]. This heterogeneity raises important questions regarding tumor histogenesis. One proposed mechanism involves divergent differentiation from a common multipotent progenitor cell within the urachal epithelium, while an alternative hypothesis suggests secondary squamous metaplasia arising within an established adenocarcinoma under chronic inflammatory conditions [11,22]. The latter hypothesis may be particularly relevant in patients with bladder exstrophy, where prolonged epithelial injury could favor squamous lineage commitment.

**Figure 4:**
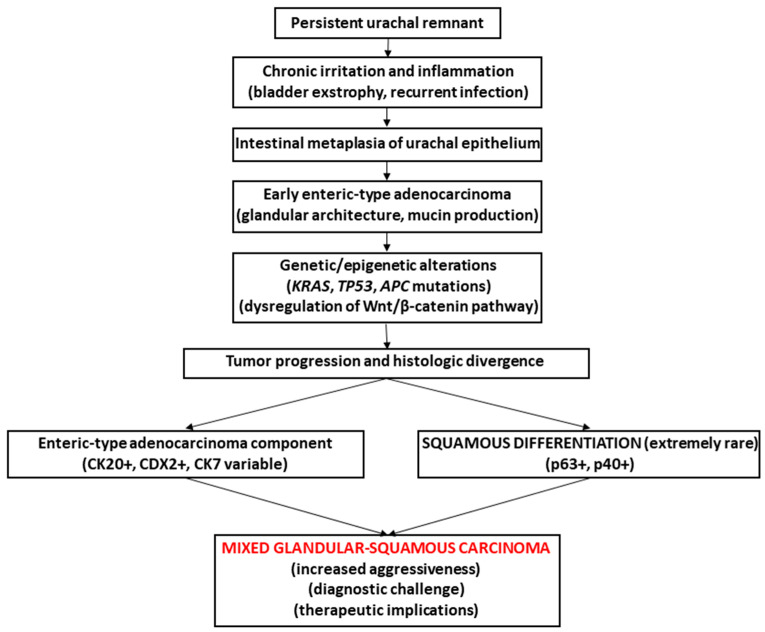
Proposed pathogenetic model of urachal carcinoma with mixed elements, enteric-type adenocarcinoma and squamous cell carcinoma.

This morphologic heterogeneity suggests complex mechanisms of tumor evolution and phenotypic plasticity in UrC. Although the precise pathways underlying squamous differentiation remain uncertain, chronic inflammation and long-standing epithelial injury are thought to play a central role [22]. Based on the clinicopathologic findings of the present case and previously reported observations, a schematic representation of the possible pathogenetic sequence leading to mixed glandular-squamous UrC is illustrated in Figure 4.

From a diagnostic standpoint, UrC with mixed glandular and squamous differentiation poses significant challenges. The differential diagnosis includes primary bladder adenocarcinoma with squamoid features, primary squamous cell carcinoma of the bladder, and metastatic adenocarcinoma from the gastrointestinal tract with secondary involvement of the bladder. Accurate diagnosis therefore requires strict adherence to established diagnostic criteria, including tumor location, growth pattern, absence of cystitis glandularis, and exclusion of an alternative primary malignancy. In the current case, the presence of bladder exstrophy as a strong predisposing factor, combined with appropriate morphologic features and immunohistochemical confirmation of urachal origin, supports the diagnosis of primary UrC.

Over the past decades, the diagnostic and staging framework for UrC has undergone substantial refinement, reflecting increased awareness of its distinct clinicopathologic features and the need to reliably distinguish it from other primary or secondary bladder malignancies. Several diagnostic algorithms and staging systems have been proposed, each addressing specific limitations of earlier approaches [1,22,25,26,27,28,29,30]. A summary of the historical evolution of diagnostic and staging criteria for UrC is provided in Table 2.

The diagnosis of urachal carcinoma in the present case fulfills established diagnostic criteria [29]. The tumor was located in the midline at the bladder dome and arose along the urachal tract, with its epicenter outside the bladder mucosa. Adjacent bladder mucosa did not demonstrate urothelial carcinoma in situ or extensive cystitis glandularis/cystica. In addition, clinical and radiologic evaluation did not reveal another primary adenocarcinoma, supporting a primary urachal origin.

Immunohistochemistry plays a crucial role in resolving these diagnostic dilemmas. Enteric-type UrC typically expresses intestinal markers such as CK20 and CDX2, with variable CK7 expression, while lacking urothelial markers. Squamous differentiation in urachal carcinoma can be supported by immunohistochemical markers such as p40, p63, or CK5/6. In the present case, the squamous component demonstrated immunoreactivity for p63, further supporting the presence of a true squamous carcinoma component. Importantly, immunohistochemical exclusion of other primary sites, particularly colorectal carcinoma, remains essential in establishing the diagnosis [20].

The clinical implications of squamous differentiation in UrC are not well defined due to the paucity of reported cases. Limited data suggest that tumors with mixed histology may exhibit more aggressive behavior, although this observation is confounded by advanced stage at presentation in many cases. Given the already poor prognosis associated with UrC, recognition of adverse histologic features may have prognostic relevance and could influence therapeutic strategies, particularly in borderline or locally advanced disease [22].

A particularly interesting aspect of the present case is the discordant cell-cycle regulatory profile observed between the glandular and squamous components, particularly with respect to p53 and p16 expression. The enteric-type adenocarcinoma displayed a null-pattern p53 immunophenotype, which may be compatible with TP53 alteration [31,32], whereas the squamous component showed a wild-type staining pattern. This difference may suggest possible molecular divergence between the two tumor components; however, such an interpretation should be approached cautiously in the absence of molecular studies. The markedly elevated Ki-67 index in the glandular component indicates increased proliferative activity within this component. The glandular component also demonstrated focal block-type p16 staining, whereas the squamous component was negative. In non-HPV-related malignancies, p16 overexpression is generally interpreted as a marker of RB pathway dysregulation rather than viral oncogenesis [32]. In the present case, these immunohistochemical differences may reflect biological heterogeneity within the tumor; however, definitive conclusions regarding tumor evolution cannot be drawn without molecular analysis. From a translational perspective, characterization of *TP53* and *RB* pathway status could have future therapeutic relevance, particularly as targeted strategies addressing cell-cycle dysregulation continue to evolve. Management of UrC remains primarily surgical, with en bloc resection of the tumor, urachus, and umbilicus forming the cornerstone of treatment [14]. The role of adjuvant therapy is not standardized and is often extrapolated from treatment protocols for colorectal adenocarcinoma or bladder cancer [14,33]. In tumors with unusual histologic features such as squamous differentiation, optimal management remains undefined, highlighting the importance of detailed case documentation and accumulation of comparable cases in the literature.

This case contributes to the limited evidence regarding UrC with squamous differentiation and reinforces the concept that UrC represents a morphologically and biologically heterogeneous group of tumors. The association with bladder exstrophy further emphasizes the role of congenital anomalies and chronic inflammation in urachal carcinogenesis. Comprehensive histopathologic evaluation, combined with careful correlation, is essential for accurate diagnosis and appropriate management of these rare tumors.

## 4. Conclusions

The present case documents a rare example of urachal carcinoma with combined enteric and squamous differentiation occurring in the setting of bladder exstrophy. This association reinforces the role of congenital urinary tract anomalies and chronic epithelial injury in promoting neoplastic transformation within urachal remnants. The identification of a squamous carcinoma component highlights the capacity for phenotypic divergence in urachal tumors and adds to the limited reported cases with mixed morphology. Accurate classification of such tumors relies on careful correlation of clinical background, anatomic localization, morphologic features, and immunohistochemical findings.

Recognition of unusual differentiation patterns is essential to avoid diagnostic pitfalls and to improve understanding of urachal tumor pathobiology. Continued reporting of similar cases will be critical in refining diagnostic frameworks and elucidating the mechanisms underlying histologic heterogeneity in urachal carcinomas.

## Figures and Tables

**Table 1:** Reported cases of urachal carcinoma with squamous component in the English literature to date.

Author (Year)	Age/Gender	Predisposing Factors	Histological Pattern	Immunophenotype	Outcome (at the Time of Manuscript Publication)
Sheldon et al. (1984) [1]	62/male	Non reported	Adenocarcinoma with focal squamous differentiation	CK20+, CK7 focal	Died of disease
Siefker-Radtke et al. (2003) [21]	55/male	Chronic cystitis	Enteric adenocarcinoma with squamous areas	CDX2+, p63+	Alive with disease
Paner et al. (2012) [11]	68/female	Not reported	Mixed glandular-squamous carcinoma	CK20+, p40+	Died of disease
Gopalan et al. (2015) [22]	60/male	Bladder anomaly	Adenocarcinoma with keratinizing squamous component	CDX2+, p63+	Recurrence
Present case	53/male	Bladder exstrophy	Enteric adenocarcinoma with squamous carcinoma	CK20+, CDX2+, beta-catenin+ (membrane), p53 mutant (null-type), p16 focal block-type staining, GATA3-	Alive with disease

**Table 2:** Evolution of diagnostic and classification criteria for urachal carcinoma.

Classification, Year	Main Diagnostic Criteria	Key Features/Limitations
Wheeler et al., 1954 [25]	Bladder tumor with mucinous features arising in the midline	Early descriptive anatomic-pathologic observations
Mostofi et al., 1955 [26]	Mucinous adenocarcinoma of the bladder with intestinal-type morphology	Introduced histologic features resembling colorectal carcinoma
Sheldon et al., 1984 [1]	Tumor located at bladder dome or anterior wall; predominant extravesical growth; absence of cystitis cystica/glandularis; exclusion of primary adenocarcinoma elsewhere	First comprehensive diagnostic and staging system; complex and sometimes difficult to apply
Johnson et al., 1985 [27]	Similar anatomic and exclusion criteria to Sheldon	Minor refinements; limited reproducibility
Gopalan et al., 2009 [22]	Emphasis on midline location, deep epicenter, and exclusion of metastatic disease	Reinforced anatomic and histologic principles
Dhillon et al., 2015 [28]	Incorporation of histologic subtypes and cytologic features	Highlighted morphologic heterogeneity
WHO classification of Tumors of the Urinary System [29]	Glandular urachal carcinoma: tumor at dome/midline; sharp demarcation from surface urothelium; epicenter in muscularis or beyond; absence of extensive cystitis glandularis/cystica; absence of urothelial CIS; exclusion of another primary adenocarcinoma. Non-glandular urachal carcinoma: tumor at dome/anterior wall; deep epicenter; association with urachal remnants; exclusion of another primary carcinoma	Established urachal carcinoma as a distinct entity; no dedicated WHO staging system
Dublin International Society of Urological Pathology (ISUP) Consensus Conference—Best Practice Recommendations, 2025 [30]	Diagnosis based on integrated clinical, radiologic, and pathologic correlation; application of WHO 2022 criteria for both glandular and non-glandular urachal carcinomas [29]; careful gross examination and standardized sampling; exclusion of metastatic disease; immunohistochemistry used selectively, particularly in TUR specimens; classification of non-glandular urachal carcinomas aligned with bladder carcinoma principles.	Most recent expert consensus; focuses on practical diagnostic guidance, reporting standards, and harmonization rather than proposing a new independent classification system

## Data Availability

No new data were created for this case report.
